# Case report: postpartum cerebral venous thrombosis misdiagnosed as postdural puncture headache

**DOI:** 10.1186/s12871-020-00992-1

**Published:** 2020-04-07

**Authors:** Mi K. Oh, Jae H. Ryu, Woo J. Jeon, Chang W. Lee, Sang Y. Cho

**Affiliations:** 1 Department of Anesthesiology and Pain Medicine, Hanyang University Guri Hospital, Guri-si, Gyeonggi-do Republic of Korea; 2 Department of Anesthesiology and Pain Medicine, Hanyang University Guri Hospital, Guri-si, Gyeonggi-do Republic of Korea; 3 Department of Anesthesiology and Pain Medicine, Hanyang University Guri Hospital, Guri-si, Gyeonggi-do Republic of Korea; 4 Department of Anesthesiology and Pain Medicine, Hanyang University Guri Hospital, Guri-si, Gyeonggi-do, Republic of Korea; 5 Department of Anesthesiology and Pain Medicine, Hanyang University Guri Hospital, 249-1, Gyomun-dong, Guri-si, Gyeonggi-do 471-701 Republic of Korea

**Keywords:** Cerebral venous thrombosis, Postdural puncture headache, Pregnancy

## Abstract

**Background:**

Cerebral venous thrombosis can be a fatal complication of the postpartum period. Pregnancy is known to be a risk factor for thromboembolism in itself.

**Case presentation:**

A normal spontaneous vaginal delivery was planned for a 20-year-old primigravida patient with patient-controlled epidural analgesia. Next morning, the patient complained of an occipital headache. An epidural blood patch was performed for diagnostic and therapeutic purpose with 10 ml of autologous blood. That night, she had an episode of seizures. Endotracheal intubation was done to secure the airway. She was transferred to an intensive care unit. Brain CT angiography and MRI showed superior sagittal sinus thrombosis with acute infarct and mild subarachnoid haemorrhage. For cerebral venous thrombosis treatment, heparin was injected and for intracranial pressure control, a hypertonic solution was injected. Despite this medical treatment, intracranial pressure continued to rise. The next day, her mental state changed to stupor. Emergency decompressive craniectomy was performed. Her mental state improved rapidly after surgery. A week later, she was transferred to a general ward. Her health recovered and she was discharged.

**Conclusions:**

We experienced postpartum cerebral venous thrombosis misdiagnosed as postdural puncture headache. We hope that this case report would be helpful in situation which a postpartum young woman complains severe headache in spite of management for headache including autologous epidural blood patch.

## Background

Cerebral venous thrombosis (CVT) can be a fatal complication of the postpartum period [1]. Pregnancy is known to be a risk factor for thromboembolism in itself. Because the most common symptom of CVT is a non-specific headache, it is difficult to diagnose. We report a case of a CVT patient who was misdiagnosed with postdural puncture headache.

### Case presentation

A 20-year-old primigravida patient was referred to our hospital with premature rupture of membrane at 35 weeks of gestation. She has no other medical history except that she was a hepatitis B virus carrier. In the blood test on admission, coagulation profile including prothrombin time (INR) (0.98), activated partial thromboplastin time (25 s) and platelet count (295,000/mm^3^) were within normal limit. A normal spontaneous vaginal delivery was planned with patient-controlled epidural analgesia. With the patient in the left lateral decubitus position, a 17-gauge Tuohy needle was inserted at the L4–5 interspace. The epidural space was confirmed with loss of resistance technique on the second attempt. A 19-gauge epidural catheter was inserted through the needle. On aspiration, there was no cerebrospinal fluid or blood. Then, an initial loading dose of 0.125% levobupivacaine (9 ml) and 50 micrograms of fentanyl was injected through the epidural catheter. The background infusion rate was 4 ml/h with 0.0625% levobupivacaine and self-administered 5 ml boluses at intervals of 10 min. Two hours later, she delivered a 2.38 kg female. The Apgar score of the infant at 1 min was 7 and at 5 min was 9.

Next morning, the patient complained of an occipital headache. The pain was worse when she sat down, but did not improve when she lay down. There were too many discrepancies to diagnose postdural puncture headache. There was no definite evidence of dural puncture and the symptoms were not specific. However, there was a possibility of an unrecognized dural puncture and symptoms of postdural puncture headache can vary from patient to patient. After consult with obstetrician and written informed consent by her husband, the epidural blood patch for diagnostic and therapeutic purpose with 10 ml autologous blood was performed. The patient said that symptoms were somewhat improved. At that time, she was diagnosed with postdural puncture headache.

**Fig. 1:**
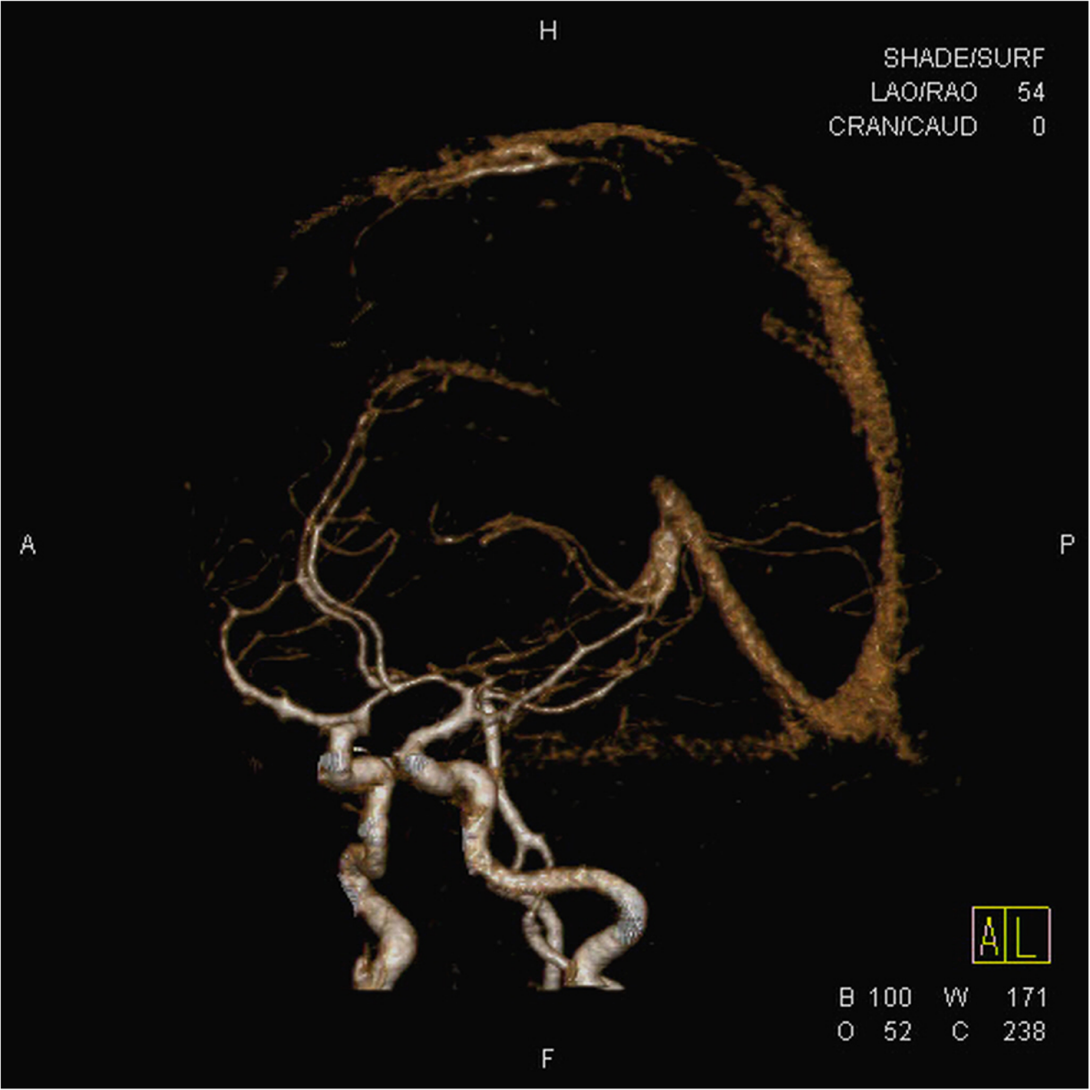
MRI image showed superior sagittal sinus thrombosis with acute infarct and mild subarachnoid haemorrhage

**Fig. 2:**
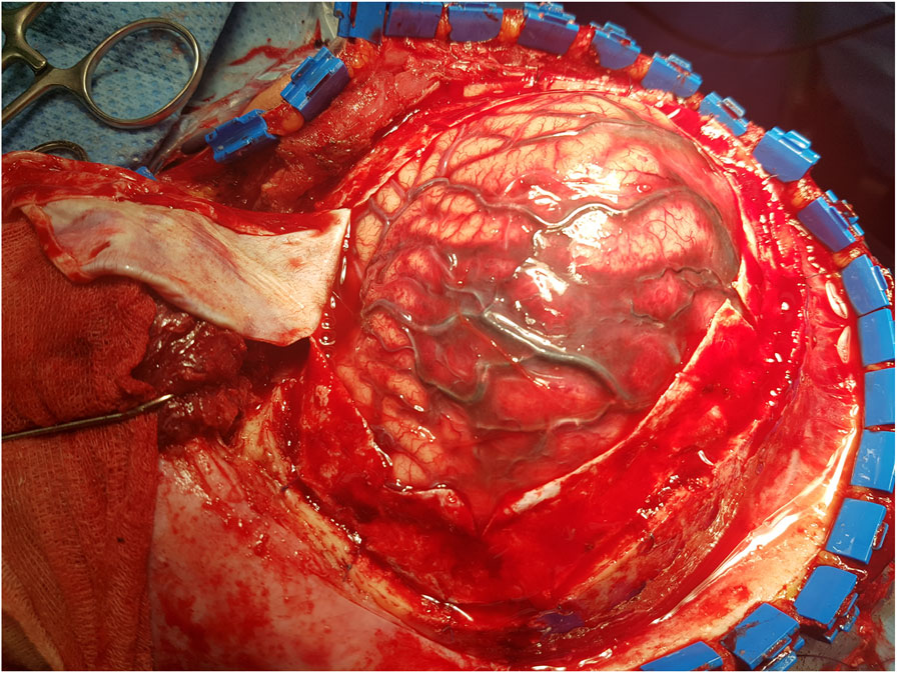
Photograph of operation field. There were severe cerebral edema and venous thrombosis

That night, she had an episode of seizures. Endotracheal intubation was done to secure the airway. She was transferred to an intensive care unit. Magnetic resonance image (MRI) showed superior sagittal sinus thrombosis with acute infarct and mild subarachnoid haemorrhage (Fig. 1). For CVT treatment, low molecular weight heparin (enoxaparin sodium, Cnoxane, 60 mg) was injected subcutaneously two times during one day and for intracranial pressure control, an osmotic diuretics (Cerol) was injected. Despite these medical treatments, intracranial pressure continued to rise. The next day, her mental state changed to stupor. Brain CT showed diffuse brain swelling and aggravating venous infarct. An emergency decompressive craniectomy was performed. During the surgery, severe brain oedema and venous thrombosis were noted. (Fig. 2). After surgery, low molecular weight heparin was continuously administered with previous method during 10 days and monitoring aPTT according to neurologist consultation. Her mental state improved rapidly after the operation. A week later, she was transferred to a general ward. After that, apart from some neurological sequelae and right-side motor weakness, her health recovered and she was discharged.

## Discussion and conclusions

Cerebral venous thrombosis can be a fatal complication of the postpartum period. The incidence of venous thrombosis during pregnancy or in the puerperium has been reported to vary from 0.018 to 0.2% depending on the study [1–3]. Because 13.4% of CVT patients are known to be at an increased risk of an unfavourable outcome [4], it is important to diagnose and treat the condition correctly.

During pregnancy, coagulation factors I, II, VII, VII, IX and XII increase [5]. In addition, physiologically, oestrogen contributes to vein expansion and congestion. Venous stasis results in a coagulation enhanced state in which thrombosis is likely to occur. Virchow’s three signs (hypercoagulation, venous congestion, and tissue damage) reduce the risk of bleeding during labour, but they increase the risk of thromboembolism in the puerperium. Other risk factors for thrombosis include postpartum haemorrhage, varicose veins, caesarean section, obesity, and history of thromboembolic disease at previous pregnancy, preeclampsia, associated malignancy, a genetic defect of coagulation inhibitor, anaemia (< 9.9 g/dL) and placental abruption [4, 6, 7]. A large population-based cohort study [6] reported that risk of venous thromboembolism was peak during the first 3 week postpartum and women in their third trimester have 6 fold risk than their time outside trimester. Preeclampsia, hypertensive disorders of pregnancy, can progress to eclampsia, which is characterized by seizure activity. Preeclampsia associated with posterior reversible encephalopathy syndrome may be differentially diagnosed with CVT [8]. In this case, CVT appeared without any other risk factors except pregnancy.

Diagnosis of CVT is difficult. In particular, the differential diagnosis between CVT and postdural puncture headache can be very difficult. In the USA, about 61% of patients who undergo normal spontaneous vaginal delivery use epidural analgesia [9]. The incidence of postdural puncture headache is estimated to be between 30 and 50% following diagnostic or therapeutic lumbar puncture, 0–5% following spinal anaesthesia and up to 81% following accidental dural puncture during epidural insertion in the pregnant woman. Symptoms of CVT include papilledema, seizures, focal sensory or motor signs, aphasia, psychiatric disturbances, and cranial nerve palsies, but headache is common as an early symptom [10]. The incidence of CVT is much lower than postdural puncture headache. Therefore, patients who complain of postpartum headache are more likely to be diagnosed with postdural puncture headache even if they have CVT, such as in this case. Because the timing of the diagnosis is important in the prognosis of CVT, this delay can be fatal. Therefore, it is necessary to closely observe symptoms in patients who complain of postpartum headache. Then, if CVT is suspected, it is important to perform image studies, such as contrast-enhanced MRI and CT, without delay [11].

On the other hand, CVT is known to be associated with dural puncture [12, 13]. Dural puncture can result in low CSF pressure, which can affect the brain blood vessels and sinuses if the brain shifts down. This can lead to venous wall deformation which can induce thrombosis. As we mentioned above, it is difficult to distinguish between a headache due to postdural puncture and that due to CVT. In general, a dural puncture headache improves when the patient lies down and is completely resolved by an epidural blood patch. However, if it is accompanied by CVT, headaches may recur or more serious symptoms may occur later. Therefore, in patients who have had a dural puncture, if the symptoms are ambiguous or if the headache continues after the blood patch, CVT should be considered.

The treatment of CVT can be divided into anticoagulant therapy and symptomatic treatment including control of seizure and elevated intracranial pressure [13]. Anticoagulant therapy can avoid thrombus extension. Subcutaneous LMWH, intravenous heparin or oral anticoagulation are all known to be useful. Systemic or local thrombolytic therapy is not recommended. Approximately 40–50% of CVT patients also have intracranial cerebral haemorrhage, and these anticoagulant therapies may worsen that [4]. However, it is recommended that anticoagulant treatment is continued in patients with CVT even in the presence of intracranial cerebral haemorrhage [13]. Also, patient with CVT should be treated either dose-adjusted intravenous heparin or with body-weight –adjusted subcutaneous LMWH with monitoring activated partial thromboplastin time (at least double time) and INR (goal of 2–2.5) [13].

Cerebral venous thrombosis is one of the rare complications of the postpartum period. Because CVT is known to be related to an unfavourable outcome, it is very important to diagnose and treat it correctly. However, it is difficult to diagnose because the symptoms of CVT are not specific. If the patient has many risk factors associated with the CVT and shows symptoms, it is important to use appropriate diagnostic methods, such as contrast-enhanced CT or MRI.

In conclusion, we experienced postpartum cerebral venous thrombosis misdiagnosed as postdural puncture headache. We hope that this case report would be helpful in situation which a postpartum young woman complains severe headache in spite of management for headache including autologous epidural blood patch.

## Data Availability

Not applicable.

## Abbreviations

CVT: Cerebral venous thrombosis
MRI: Magnetic resonance image
